# Enhancement of SARS-CoV-2 N Antigen-Specific T Cell Functionality by Modulating the Autophagy-Mediated Signal Pathway in Mice

**DOI:** 10.3390/v15061316

**Published:** 2023-06-02

**Authors:** Ziyu Wen, Yue Yuan, Yangguo Zhao, Haohang Wang, Zirong Han, Minchao Li, Jianhui Yuan, Caijun Sun

**Affiliations:** 1 School of Public Health (Shenzhen), Shenzhen Campus of Sun Yat-Sen University, Shenzhen 518107, China; wenzy3@mail2.sysu.edu.cn (Z.W.); yuany263@mail2.sysu.edu.cn (Y.Y.); zhaoyg3@mail2.sysu.edu.cn (Y.Z.); wanghh7@mail2.sysu.edu.cn (H.W.); hanzr@mail2.sysu.edu.cn (Z.H.); liminchao@mail2.sysu.edu.cn (M.L.); 2 Nanshan District Center for Disease Control and Prevention, Shenzhen 518000, China; 3 Key Laboratory of Tropical Disease Control (Sun Yat-Sen University), Ministry of Education, Guangzhou 510080, China

**Keywords:** SARS-CoV-2, N protein, autophagy, T cellular immunity

## Abstract

The frequent SARS-CoV-2 variants have caused a continual challenge, weakening the effectiveness of current vaccines, and thus it is of great importance to induce robust and conserved T cellular immunity for developing the next-generation vaccine against SARS-CoV-2 variants. In this study, we proposed a conception of enhancing the SARS-CoV-2 specific T cell functionality by fusing autophagosome-associated LC3b protein to the nucleocapsid (N) (N-LC3b). When compared to N protein alone, the N-LC3b protein was more effectively targeted to the autophagosome/lysosome/MHC II compartment signal pathway and thus elicited stronger CD4^+^ and CD8^+^ T cell immune responses in mice. Importantly, the frequency of N-specific polyfunctional CD4^+^ and CD8^+^ T cells, which can simultaneously secrete multiple cytokines (IFN-γ^+^/IL-2^+^/TNF-α^+^), in the N-LC3b group was significantly higher than that in the N alone group. Moreover, there was a significantly improved T cell proliferation, especially for CD8^+^ T cells in the N-LC3b group. In addition, the N-LC3b also induced a robust humoral immune response, characterized by the Th1-biased IgG2a subclass antibodies against the SARS-CoV-2 N protein. Overall, these findings demonstrated that our strategy could effectively induce a potential SARS-CoV-2 specific T cellular immunity with enhanced magnitude, polyfunctionality, and proliferation, and thus provided insights to develop a promising strategy for the design of a novel universal vaccine against SARS-CoV-2 variants and other emerging infectious diseases.

## 1. Introduction

The pandemic of coronavirus disease 2019 (COVID-19), caused by severe acute respiratory syndrome coronavirus 2 (SARS-CoV-2), has continued to threaten global public health [1]. The COVID-19 vaccine, as the most powerful weapon to control this pandemic, has been extensively developed, and at least 15 kinds of COVID-19 vaccines have been approved for clinical use by the World Health Organization (WHO), including inactivated vaccines, protein subunit vaccines, mRNA vaccines, and viral vector vaccines [2,3]. So far, these vaccines are mainly targeted to the S protein, which contains two subunits S1 and S2 that contribute to viral attachment, fusion, and entry, to induce neutralizing antibodies [4]. However, the frequent emergence of SARS-CoV-2 variants, such as Delta and Omicron, has greatly weakened vaccine effectiveness and caused breakthrough infections frequently due to the waned neutralizing antibody titers and the low frequency of virus-specific memory B cells [5,6,7]. Alternatively, it is of great importance to induce robust and conserved T cell-mediated immunity for developing the next-generation vaccine against SARS-CoV-2 variants.

Recent studies have revealed that the T cell immune responses play a critical role in controlling viral replication [6,8]. For example, a high frequency of SARS-CoV-2 specific T cells was identified in COVID-19 convalescent individuals [9,10]. Importantly, when compared to antibody responses, T cell immune responses are usually more conserved against viral variants [11,12,13]. Furthermore, the memory T cells usually have a long-term survival time [8]. For example, one study showed that the memory T cells can persist for more than 17 years in some convalescent individuals from SARS-CoV infection [14], and at least 20 months in some individuals who have recovered from SARS-CoV-2 infection [8]. Besides seropositive patients, the individuals with asymptomatic or mild disease courses of COVID-19 also had abundant memory T cell responses [15]. Thus, it is worth studying the possibility to develop a long-lasting universal COVID-19 vaccine by targeting broadly cross-reactive T cell epitopes.

The nucleocapsid (N) protein is conserved with approximately 90% amino acid homology between various SARS-CoV-2 variants, and it contains some cross-reactive T cell epitopes [16,17,18]. Therefore, it is expected to be a promising target for the universal COVID-19 vaccine. Previous studies demonstrated that the N-based vaccine generated partial protection against the SARS-CoV-2 challenge, and also enhanced the protection efficacy when combined with the S-antigen-based COVID-19 vaccine [19,20]. However, the immunogenicity of the natural N protein is relatively weak, and thus it can only elicit insufficient T cell immunity. Consequently, it is necessary to explore a novel strategy to improve the immunogenicity of the N antigen-based COVID-19 vaccine.

Autophagy, particularly macroautophagy, is a powerful tool that the host’s cells use to defend against viral infections [21]. Autophagy contributes to the delivery and processing of endogenous antigens to MHC class II molecules by the cross-presentation mechanism [22,23]. The microtubule-associated protein 1 light chain 3 beta (LC3b) is one of the key components involved in macroautophagy and is usually dispersed throughout the cytoplasm in diffuse form (LC3-Ⅰ). Upon the formation of the autophagosome, LC3-I is converted to the phosphatidylethanolamine-coupled LC3-II form, which can then become the punctate form at the autophagosome [24]. The recruitment of LC3-II is critical for the subsequent regulation of adaptive immune responses by the autophagosome/lysosome/MHC II compartment signal pathway. Actually, our previous work showed that when the Gag antigen of the simian immunodeficiency virus (SIV) was fused to the LC3b protein, it effectively enhanced the SIV antigen-specific T cell immunity with magnitude and polyfunctionality [25]. Based on this finding, we further verified this strategy to enhance the immunogenicity of the SARS-CoV-2 N antigen in the present study, and thus provided insights for the development of a T cell-based universal vaccine against SARS-CoV-2 variants.

## 2. Materials and Methods

### 2.1. Construction of DNA Vaccine

The sequences of the SARS-CoV-2 N gene and mouse LC3b gene were obtained from the National Center for Biotechnology Information (NCBI) and we conducted codon optimization for efficient expression in mammalian cells. The fusion gene, N-LC3b, was obtained by the overlap PCR method (Table S1). Specifically, the C terminal of the N gene was fused with the N terminal of the LC3b gene bridged with the GGGSGGG linker, and the Flag Tag was added to the C terminal of the fusion gene. Subsequently, the LC3b, the N, and the fused gene N-LC3b were cloned into the pVAX1 expression vector (Invitrogen, Carlsbad, CA, USA).

### 2.2. Western Blotting Analysis

293T cells (from human embryonic kidney cells) and Hela cells (from human cervical cancer cells) were cultured in complete Dulbecco’s modified Eagle’s medium (DMEM, Gibco, New York, NY, USA) containing 10% fetal bovine serum (FBS, Gibco, New York, NY, USA) and 1% penicillin/streptomycin (Gibco, New York, NY, USA), at 37 °C in an atmosphere of 5% of CO_2_. To verify the protein expression, 293T cells were seeded at a density of 5 × 10^5^ cells in 6-well plates and transfected with the corresponding plasmids using Lipofectamine 2000 reagent (Invitrogen, Carlsbad, CA, USA) in OptiMEM serum-free medium for 24 h. To detect whether the decrease in N-LC3b was correlated with autophagy, Hela cells were transfected with pVAX-N, pVAX-N-LC3b, respectively, for 6 h and treated with rapamycin (RAPA) (100 nM) or chloroquine (CQ) (75 μM) for 24 h. Cells were lysed using RIPA cell lysis buffer (Beyotime, Shanghai, China) and SDS-PAGE was conducted. After the protein was transferred to the nitrocellulose filter (NC) membrane, anti-SARS-CoV-2 N antibody (CST, Boston, MA, USA), anti-LC3b antibodies (Sigma, Darmstadt, Germany), and anti-GAPDH antibody (Abmart, Shanghai, China) were incubated overnight at 4 °C. Next, the membrane was incubated with the horseradish peroxidase (HRP)-conjugated anti-rabbit IgG antibody or anti-mouse IgG antibody (Abclonal, Wuhan, China), and detected with a chemiluminescent HRP substrate (Tanon, Shanghai, China).

### 2.3. Confocal Microscopy

DC2.4 cells (from murine bone marrow-derived dendritic cells) were cultured in complete Roswell Park Memorial Institute 1640 medium (RPMI 1640, Gibco), containing 10% fetal bovine serum and 1% penicillin/streptomycin, at 37 °C in an atmosphere of 5% of CO_2_. Hela cells or DC2.4 cells were seeded on microscope cover glasses and transfected with pVAX-N-LC3b or pVAX-N for 36 h, then treated with or without chloroquine (CQ) for 12 h. Cells were washed with phosphate-buffered saline (PBS) three times and fixed in 4% paraformaldehyde (Beyotime, Shanghai, China) for 1h at 4 °C. Next, cells were permeabilized and blocked with QuickBlock™ Blocking Buffer for Immunol Staining reagents (Beyotime, Shanghai, China) for 15 min at room temperature. Then, the cells were washed with PBST (PBS supplemented with Tween-20) and incubated with corresponding primary antibodies in 1% bovine serum albumin solution at a dilution of 1:200 overnight at 4 °C. After being washed with PBST, secondary antibodies were added and incubated for 2 h. Finally, the cells were stained with DAPI nucleic acid stain (1 mg/mL, Beyotime, Shanghai, China) for 15 min and imaged using a confocal microscope (Zeiss, Oberkochen, Germany).

### 2.4. Animal Immunization

Female, six-week-old, non-pathogenic BALB/c mice were raised in an SPF environment in this study. Twenty mice were randomly allocated to four groups, including the negative control group (PBS and pVAX-LC3b), and the experimental groups (pVAX-N and pVAX-N-LC3b). All mice were injected with 0.5% bupivacaine hydrochloride (Sigma, Darmstadt, Germany) in the gastrocnemius muscle of each hind leg three days before the initial immunization. On days 0 and 14, 50 μg of the corresponding DNA plasmid dissolved in 100 μL of sterile PBS was injected into the gastrocnemius muscle of both hind legs of the mice (50 μL for each leg). On day 28, mouse serum was collected for antibody detection by the enzyme-linked immunosorbent assay (ELISA). Mouse spleen lymphocytes were harvested and subjected to enzyme-linked immunosorbent spot (ELISPOT) assay, intracellular cytokine staining (ICS) assay, and carboxyfluorescein diacetate succinimidyl ester (CFSE)-based proliferation assay.

### 2.5. ELISA

SARS-CoV-2 nucleocapsid-specific antibodies were detected by ELISA as in our previously reported method [26,27]. In brief, 96-well EIA plates (Corning Inc, Corning, NY, USA) were coated with 100 ng per well of SARS-CoV-2 N protein antigens (Abclonal, Wuhan, China) in PBS overnight at 4 °C. Plates were washed with PBST three times and then blocked with 5% skimmed milk in PBST for 1 h at 37 °C. Mouse serum was diluted at 1:25, and then added to the washed plates and incubated for 2 h at 37 °C. After washing, plates were incubated with a 1:5000 dilution of HRP conjugated anti-mouse IgG secondary antibody (Abcam, Cambridge, UK), and HRP conjugated anti-mouse IgG1 secondary antibody (Abcam), HRP conjugated anti-mouse IgG2a secondary antibody (Proteintech, Wuhan, China), and HRP conjugated anti-mouse IgG2c secondary antibody (Abcam, Cambridge, UK), respectively, for 1 h at 37 °C. The color reaction was substrated with 3,3′,5,5′-Tetramethylbenzidine (TMB) and stopped with 1 M H_2_SO_4_. The value was read at a 450 nm wavelength using a Synergy HT Multi-Mode Plate Reader (BioTek, Winooski, VT, USA).

### 2.6. IFN-γ ELISPOT Assay

Interferon Gamma (IFN-γ) ELISPOT was performed using freshly isolated mouse splenic lymphocytes as previously described [28,29]. In brief, PVDF 96-well plates (Millipore, Billerica, MA) were pre-coated with mouse IFN-γ coating antibody (U-CyTech, Utrecht, The Netherlands) overnight at 4 °C. Mouse splenic lymphocytes were isolated using a density gradient medium (Dakewe Biotech, Shenzhen, China). The N peptide pools were 15 amino acids in length and overlapped by 11 amino acids covering the full-length SARS-CoV-2 N protein, and were synthesized by GenScript company, and then dissolved in dimethyl sulfoxide (DMSO). The 2.5 × 10^5^ mouse splenocytes were plated into each well and stimulated with N peptide pool at a final concentration of 2 μg/mL for each peptide, while DMSO was performed as a mock simulation. After incubation for 24 h, the plates were incubated with biotinylated detection antibodies (U-CyTech, Utrecht, The Netherlands) and developed with alkaline phosphatase-conjugated streptavidin (U-CyTech, Utrecht, The Netherlands) and NBT/BCIP reagent (Pierce, Rockford, IL, USA). Finally, the spots were counted with an ELISPOT reader (Mabtech, Stockholm, Sweden).

### 2.7. ICS

ICS assay was performed according to our previous method [30]. Briefly, mouse splenic lymphocytes were seeded in the 96-well plates at 2 × 10^6^ per well and stimulated with the SARS-CoV-2 N peptide pool (2 μg/mL for each peptide) at 37 °C for 1.5 h. Then, brefeldin A (BD, Franklin Lakes, NJ, USA) was added and incubated for 16 h at 37 °C. The cells were harvested and stained with anti-mouse CD3-FITC, anti-mouse CD4-BB700, and anti-mouse CD8-PE cy7 (BD, Franklin Lakes, NJ, USA) for 30 min, and protected from light at room temperature. Then, the cells were fixed and permeabilized with cytofix/cytoperm (BD, Franklin Lakes, NJ, USA) for 20 min, and protected from light at 4 °C. After being washed with perm/wash (BD, Franklin Lakes, NJ, USA), the cells were stained with anti-mouse IFN-γ-APC, anti-mouse IL-2-BV605, and anti-mouse TNF-α-PE (BD, Franklin Lakes, NJ, USA) for 1 h, and protected from light at 4 °C. Finally, the cells were washed with FACS washing buffer (PBS supplement with 2% heat-inactivated FBS) three times and resuspended in PBS. The data were obtained with Beckman CytExpert software and analyzed using FlowJo software (version 10.8.1).

### 2.8. CFSE-Based Proliferation Assay

Mouse splenic lymphocytes were resuspended at 1 × 10^6^/mL in 0.1% FBS/PBS and incubated at 37 °C for 10 min with 0.2 μM CFSE (ThermoFisher, Waltham, MA, USA). An equal volume of ice-cold RPMI 1640 was added and an ice bath was run for 5 min to terminate the staining. After the addition of serum and washes with RPMI 1640, cells were resuspended at 1 × 10^6^ cells/mL and plated into 48-well U-bottom plates at 500 μL volumes and simulated with a SARS-CoV-2 N peptide pool (2 μg/mL for each peptide). After being simulated for five days, cells were harvested and washed with FACS washing buffer and stained with anti-mouse CD3-PE, anti-mouse CD4-BB700, anti-mouse CD8-PE-Cy7, and fixable viability stain 780 (BD, Franklin Lakes, NJ, USA). Cells were washed and a flow cytometric analysis was conducted using Beckman CytExpert. The data were analyzed using FlowJo software (version 10.8.1).

### 2.9. Statistical Analysis

Graphical presentations and statistical analyses were performed using GraphPad Prism software version 8. Statistical significance was calculated using ANOVA with Holm–Sidak multiple comparisons tests. * *p* < 0.05; ** *p* < 0.01; *** *p* < 0.001; **** *p* < 0.0001.

## 3. Results

### 3.1. Functional Targeting of SARS-CoV-2 N-LC3b Fusion Antigen to Autophagosomes/Lysosomes/MHC II Compartments Signal Pathway

**Figure 1:**
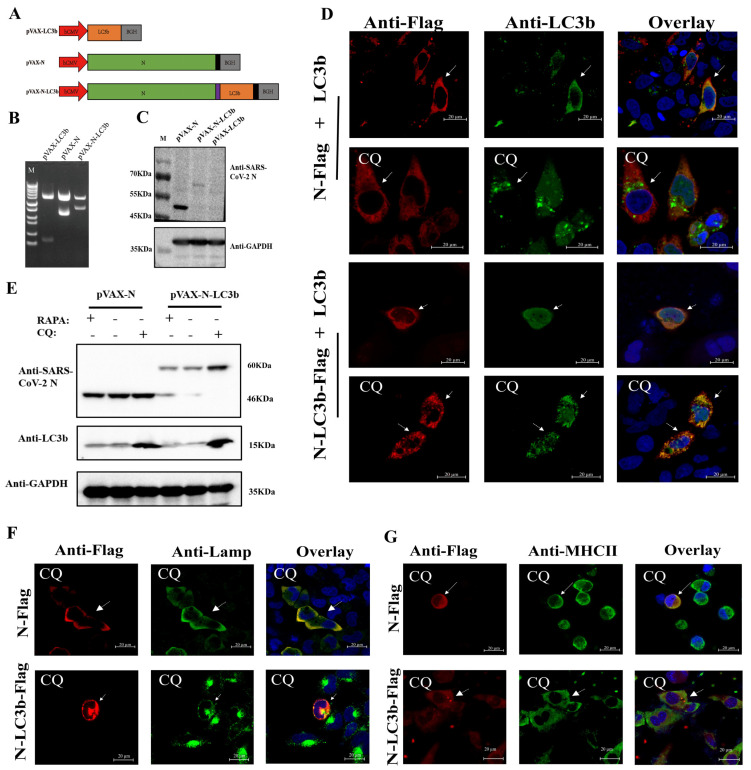
The SARS-CoV-2 N-LC3b fusion protein was effectively processed by autophagy pathway and presented to MHC II. (**A**) Schematic representation of constructs carrying various combinations of mouse LC3b gene or SARS-CoV-2 under the CMV promoter denoted as pVAX-LC3b, pVAX-N, and pVAX-N-LC3b, respectively. (**B**) The plasmids were identified by HindⅢ, XbaI digestion. Lane1: DNA marker, 10,000, 8000, 6000, 5000, 4000, 3000, 2000, 1000, 750, 500, 250, 100 bp. Lane2: pVAX-LC3b; Lane3: pVAX-N; Lane4: pVAX-N-LC3b. (**C**) The protein expression was detected using anti-SARS-CoV-2 N antibodies and anti-GAPDH antibodies, respectively. The molecular weight of N protein: 46KDa; the molecular weight of N-LC3b fusion protein: 60KDa. (**D**) HeLa cells were co-transfected with pVAX-LC3b and pVAX-N or pVAX-N-LC3b plasmid with or without CQ (75 μM) treatment, and then stained with rabbit anti-LC3b IgG antibody and mouse anti-Flag IgG antibody. Subsequently, Cy3-labeled goat anti-mouse IgG (red fluorescence) and Alexa Fluor 488-labeled goat anti-rabbit IgG (green fluorescence) secondary antibodies were used; (**E**) Hela cells were transfected with pVAX-N, and pVAX-N-LC3b, respectively, and treated with RAPA (100 nM) or CQ. The protein expression was detected using anti-SARS-CoV-2 N antibodies and anti-mouse LC3b antibodies. GAPDH blots demonstrated that the overall protein was not affected after CQ and RAPA treatment. (**F**) HeLa cells and (**G**) DC2.4 cells were transfected with pVAX-N, pVAX-N-LC3b with CQ treatment, and then stained with mouse anti-LAMP-2 IgG antibody or mouse anti-MHC II IgG antibody and rabbit anti-Flag IgG antibody. Subsequently, Cy3-labeled goat anti-rabbit IgG (red fluorescence) and Alexa Fluor 488-labeled goat anti-mouse IgG (green fluorescence) secondary antibodies were used; the nucleus was stained with DAPI (blue fluorescence). The scale bar represents 20 μm. N: nucleocapsid protein. LC3b: microtubule-associated protein 1 light chain 3 beta. RAPA: rapamycin. CQ: chloroquine. The data represent three independent experiments.

We designed and constructed a series of recombinant DNA vectors carrying the mouse LC3b gene, SARS-CoV-2 N gene, and SARS-CoV-2 N-LC3b fusion gene, respectively (Figure 1A,B). Using anti-SARS-CoV-2 N antibodies to recognize the SARS-CoV-2 N-LC3b fusion proteins, we confirmed the appropriate protein expression of these constructs by Western blotting assay (Figure 1C).

We next investigated the subcellular localization of the SARS-CoV-2 N protein in the presence or absence of LC3b protein fusion using confocal microscopy. HeLa cells were co-transfected with pVAX-LC3b, pVAX-N-Flag, or pVAX-N-LC3b-Flag, and our results showed that the autophagosomes (green puncta) were co-localized with the N-LC3b fusion protein (red puncta) after being treated with CQ, which can block the fusion of autophagosomes with lysosomes, and thereby enable the visualization of the accumulation of autophagosomes (Figure 1D, bottom). However, the location of the N protein was significantly different from that of the N-LC3b fusion protein. The N protein alone was distributed homogenously throughout the cytoplasm whether CQ existed or not, and the autophagosomes (green puncta) were not co-localized with the N protein (red puncta) (Figure 1D, top). Moreover, the expression of the N-LC3b protein was significantly decreased after RAPA treatment, which can induce autophagic flux, and it obviously increased after CQ treatment, which might be attributed to CQ blocking the autophagy-mediated degradation of the N-LC3b protein, but the expression of the N protein alone was not changed after CQ treatment or RAPA treatment (Figure 1E). Subsequently, we also found that the N-LC3b protein was co-localized to LAMP II, which is a marker of endosomes/lysosomes (Figure 1F). To further verify whether the degradation of N-LC3b could be presented to the MHC II compartments, DC2.4 cells were transfected with pVAX-N and pVAX-N-LC3b, respectively, and then subjected to an immunofluorescence assay. The results showed that the N-LC3b protein was effectively co-localized to the MHC II compartments (Figure 1G). Taken together, these data demonstrated that the N-LC3b fusion protein can be functionally targeted to autophagosomes, processed by autophagy-mediated degradation in autolysosomes/lysosomes, and then presented to MHC II compartments for eliciting the subsequent adaptive immunity.

### 3.2. Enhancement of SARS-CoV-2 N Antigen-Specific T Cell Immune Responses by the N-LC3b Fusion Antigen

**Figure 2:**
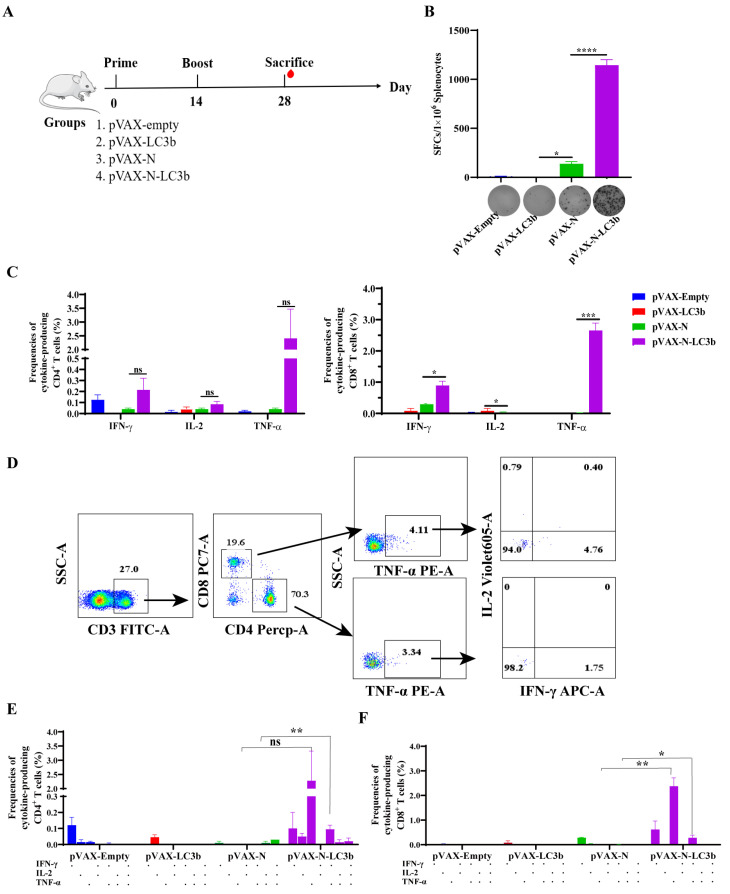
The SARS-CoV-2 N antigen-specific T cell immune responses in response to the N-LC3b fusion antigen. (**A**) The schedule of immunization experiment in mice. (**B**) The column showed the SARS-CoV-2 N-specific SFCs per 1 × 10^6^ spleen lymphocytes as measured by IFN-γ ELISPOT assay. The bottom of the graph showed the original picture for our ELISPOT assay (2.5 × 10^5^ cells per well). (**C**) The frequencies of total IFN-γ, TNF-α, or IL-2 cytokine-positive CD4^+^ T cells (**left**) and CD8^+^ T cells (**right**). (**D**) The graph represented the gating strategy of polyfunctional T cells analysis. Multiple cytokines-positive CD4^+^ T cells (**E**) and CD8^+^ T cells (**F**) were analyzed by multicolor flow cytometry. Two independent experiments for animal immunization were repeated. The data were shown as the mean ± SD for each group (*n* = 5). ns: no significance. * *p* < 0.05, ** *p* < 0.01, *** *p* < 0.001, **** *p* < 0.0001, ns: no significance.

Then, we evaluated the immunogenicity of the N-LC3b fusion antigen in vivo. Mice were randomly allocated to four groups, including pVAX-Empty, pVAX-LC3b, pVAX-N, and pVAX-N-LC3b, and the antigen-specific antibodies and T cell immune responses were detected after immunization (Figure 2A). Our results demonstrated that the N antigen-specific T cell immune responses in the pVAX-N-LC3b group were greatly enhanced compared to the pVAX-N group. Of note, 1144 specific spot-forming cells (SFCs) per 10^6^ spleen lymphocytes against SARS-CoV-2 N peptide pools were observed after the pVAX-N-LC3b immunization, which was 8.28 times higher than that in the pVAX-N group (Figure 2B). Meanwhile, the total frequencies of IFN-γ^+^ or TNF-α^+^ CD8^+^ T cells, responding with N-specific peptide pools in the pVAX-N-LC3b group, were significantly higher than those in the pVAX-N group (Figure 2C). Furthermore, the induction of polyfunctional T cells secreting multiple cytokines was also analyzed in this study (Figure 2D), and the results showed that there was a higher frequency of polyfunctional CD4^+^ T cells (Figure 2E) and CD8^+^ T cells (Figure 2F) in the pVAX-N-LC3b group than that in the pVAX-N group. Overall, these results demonstrated that the N-LC3b fusion antigen elicited a potent antigen-specific CD4^+^ T and CD8^+^ T cellular immunity, with enhanced magnitude and polyfunctionality.

### 3.3. Enhancement of SARS-CoV-2 N Antigen-Specific T Cell Proliferation by the N-LC3b Fusion Antigen

**Figure 3:**
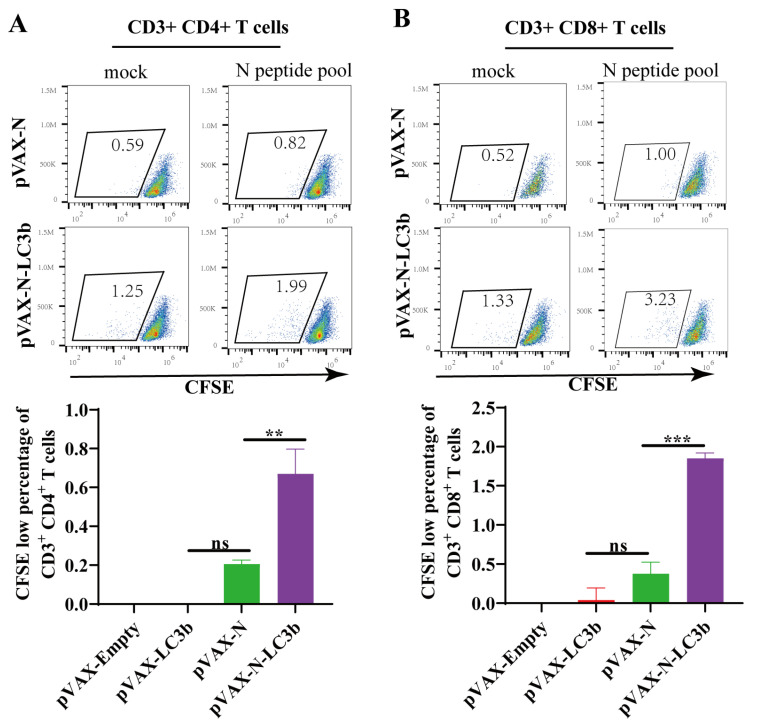
SARS-CoV-2 N antigen-specific T cell proliferation detected by CFSE-based staining. The scatter plot showed the proliferation capacity of CD3^+^ CD4^+^ T cells (**A**) and CD3^+^ CD8^+^ T cells (**B**) in response to SARS-CoV-2 N peptides stimulation or DMSO stimulation (mock). The CFSE-low cells (lower fluorescence intensity) represent these daughter cells after cell proliferation. Columns represent the percentage of N-specific CFSE-low cells in response to stimulation (subtracted by the mock). These data were shown as the mean ± SD for each group (*n* = 5). ** *p* < 0.01, *** *p* < 0.001, ns: no significance.

Recent studies demonstrated that SARS-CoV-2 specific CD4^+^ T and CD8^+^ T cells in COVID-19 convalescent individuals had strong ex vivo proliferation capacities, implying that the induction of T lymphocyte proliferation should be an important immunological parameter to evaluate an effective COVID-19 vaccine candidate. We, therefore, evaluated the SARS-CoV-2 antigen-induced proliferation capacity in response to the N-LC3b fusion antigen immunization. The fresh splenocytes from the immunized mice were labeled with CFSE and stimulated for 5 days with N-specific peptide pools, and then analyzed using flow cytometry. The percentage of CFSE low cells of both CD3^+^ CD4^+^ T cells (Figure 3A) and CD3^+^ CD8^+^ T cells (Figure 3B) in the pVAX-N-LC3b group exhibited a significant increase compared to that in the pVAX-N group. Thus, the N-LC3b fusion antigen effectively induced an improved proliferation capacity of the antigen-specific CD3^+^ CD4^+^ T and CD3^+^ CD8^+^ T lymphocytes, which is important for long-lasting immune protection.

### 3.4. Induction of Th1-Biased Immunity by the N-LC3b Fusion Antigen

**Figure 4:**
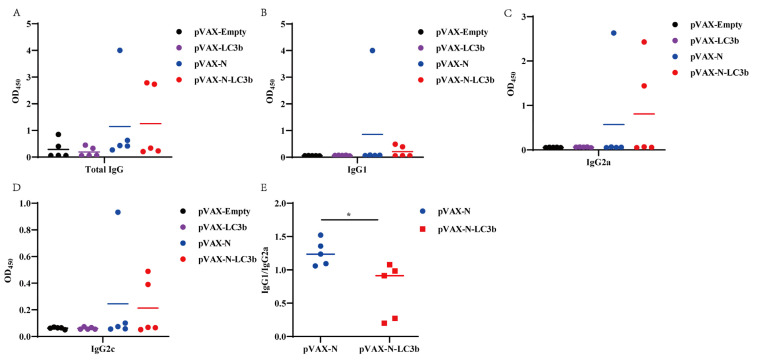
Induction of Th1-biased immunity by the N-LC3b fusion antigen. Sera (at diluted 1:25) of the immunized mice on day 28 were analyzed for the SARS-CoV-2 N specific total IgG (**A**), IgG1 (**B**), IgG2a (**C**), or IgG2c (**D**) subclass antibodies. (**E**) The ratio of IgG1/IgG2a. The data were shown as the mean ± SD for each group (*n* = 5). * *p* < 0.05.

Furthermore, we also detected the humoral immune responses elicited by our strategy. Our result showed that both the N antigen and the N-LC3b fusion antigen could effectively induce the IgG antibody immune responses (Figure 4A). Moreover, we analyzed the subclass of these IgG antibodies. In mice, IgG1 is a marker of a Th2-biased immune response, while IgG2a and IgG2c antibodies represent a Th1-biased immune response. We found that the pVAX-N immunization mainly induced the IgG1, IgG2a, and Ig2c antibodies, of which the IgG1 antibody was the most induced. However, the pVAX-N-LC3b preferentially induced the IgG2a antibody (Figure 4B–D). The ratio of IgG1/IgG2a showed that the N-LC3b fusion antigen induced a Th1-biased immune response in mice (Figure 4E).

## 4. Discussion

The accumulating data have shown that T cells play a pivotal role in fighting SARS-CoV-2 infection, and, most likely, in forming immunological memory following recovery from COVID-19 [31]. Some recovered COVID-19 patients had robust SARS-CoV-2 specific T cell immune responses but no obvious SARS-CoV-2 neutralizing antibody was detected, implying the importance of T cell immunity in controlling COVID-19 progression [15]. More importantly, T cell-mediated immunity can effectively play a critical role against viral variants, which can rapidly escape immune recognition by neutralizing antibodies [32]. T cells differentiate into effector cells by specifically recognizing the MHC-antigen peptide complex presented by antigen-presenting cells. Classically, the endogenous antigen peptides binding with MHC class I molecules are recognized by CD8^+^ T cells, while the exogenous antigen peptides degraded by the lysosomal pathway binding with MHC class II molecules are recognized by CD4^+^ T cells. In addition, antigen-presenting cells might also process exogenous antigens to CD8^+^ T cells via the MHC class I pathway but not CD4^+^ T cells, also known as cross-presentation. Interestingly, macroautophagy can have a cross-presentation function to deliver some endogenous proteins to MHC class II molecules and thus enhance the adaptive T cell immune responses [33,34]. Both our work and the other group’s study showed that the antigen design by fusing autophagy-associated protein LC3b effectively elicited a robust immune response against influenza, *Mycobacterium* tuberculosis (*Mtb*), and simian immunodeficiency virus (SIV) infections [34,35]. Consistent with these findings, we herein found that when compared to N antigen alone, the N-LC3b antigen was more effectively targeted to the autophagosome/lysosome/MHC II compartment signal pathway, and effectively elicited the stronger polyfunctional CD4^+^ and CD8^+^ T cell immune responses in mice.

Another notable observation is that the pVAX-N immunization induced a Th2-biased immune response, while the pVAX-N-LC3b immunization induced a Th1-biased immune response. An effective COVID-19 vaccine should induce the “right” antibodies and T cell responses because the “wrong” antibodies might increase the risk of immunopathologies. For example, some case reports indicated that patients with high SARS-CoV-2 IgG levels were more likely to develop severe disease [36]. Moreover, the potential risks of antibody-dependent enhancement (ADE) and enhanced respiratory disease (ERD) has been observed in animal models when immunized with some COVID-19 vaccines [37], which mainly induced the Th2-biased immune responses [38]. Although these immunized mice had significantly lower viral load after the SARS-CoV-2 challenge, they also had an eosinophilic infiltrate into the lungs, which was accompanied by ERD-related immunopathology in lung tissue [39]. Interestingly, our strategy can regulate the bias of the Th1/Th2 immune responses, as revealed by the inverted ratio of the IgG1/IgG2a subclass induced by the N-LC3b fusion antigen when compared to N antigen alone. In addition, the cytokine profile of splenocytes from the pVAX-N-LC3b immunized mice also supported a preferable Th1-biased immune response.

Our strategy induced the promising immunogenicity of the novel COVID-19 vaccine candidate in mice, but there are some limitations. For instance, we only used the DNA-based vaccine to verify this hypothesis as a model; other strategies including an mRNA-based vaccine and viral-vector-based vaccine might be more suitable to deliver this designed antigen to elicit a more robust immune response. In addition, the protective efficacy against SARS-CoV-2 infections was not evaluated in this study because of our limited resources for the high-level biosafety laboratory. In conclusion, we developed a simple and effective strategy to induce a potential SARS-CoV-2 specific T cellular immunity with enhanced magnitude, polyfunctionality, and proliferation by targeting the autophagy-mediated signal pathway, and our findings warranted a further study on protection efficacy in an animal infection model as a novel T cell-based COVID-19 vaccine candidate. In addition, this N-LC3b fusion protein is a promising component in the prime/boost vaccination strategy to elicit a balanced Th1/Th2 immunity against SARS-CoV-2 infection.

## Data Availability

Not applicable.

## Supplementary Materials

The following supporting information can be downloaded at https://www.mdpi.com/article/10.3390/v15061316/s1, Table S1: Primer Sequences.
