# Supplementary material for: Enhancement of SARS-CoV-2 N Antigen-Specific T Cell Functionality by Modulating the Autophagy-Mediated Signal Pathway in Mice
Source: Viruses. 2023 Jun 2;15(6):1316. doi: 10.3390/v15061316 (PMC10303505; doi:10.3390/v15061316)
Supplement: Supplementary file 1 [file viruses-15-01316-s001.zip › viruses-2406229-supplementary.pdf]

Supplementary Material

Supplementary Tables

Table S1. Primer Sequences

Primer	Sequence
Hind3-N-F1	CCCAAGCTTACCATGTCTGACAATGGC
Nlap-F2	TCTGCTGACTCCACCCAGGCCGGTGGTGGTTCAGGAGGAGGAATGCCGTCCGAGAAGAC
N-lap-R2	GAAGGTCTTCTCGGACGGCATTCCTCCTCCTGAACCACCACCGGCCTGGGTGGAGTCAG
Xba1-flagN-R2	GCTCTAGATTTACTTGTCATCATCATCCTTGTAGTCCACAGCCATTGCTGTCCCG
